# Efficacy of finafloxacin in a murine model of inhalational glanders

**DOI:** 10.3389/fmicb.2022.1057202

**Published:** 2022-11-24

**Authors:** Kay B. Barnes, Marc Bayliss, Carwyn Davies, Mark I. Richards, Thomas R. Laws, Andreas Vente, Sarah V. Harding

**Affiliations:** ^1^Defence Science and Technology Laboratory, Salisbury, United Kingdom; ^2^MerLion Pharmaceuticals GmbH, Berlin, Germany; ^3^Department of Respiratory Sciences, University of Leicester, Leicester, United Kingdom

**Keywords:** glanders, finafloxacin, co-trimoxazole, *Burkholderia mallei*, mouse models

## Abstract

*Burkholderia mallei*, the causative agent of glanders, is principally a disease of equines, although it can also infect humans and is categorized by the U.S. Centers for Disease Control and Prevention as a category B biological agent. Human cases of glanders are rare and thus there is limited information on treatment. It is therefore recommended that cases are treated with the same therapies as used for melioidosis, which for prophylaxis, is co-trimoxazole (trimethoprim/sulfamethoxazole) or co-amoxiclav (amoxicillin/clavulanic acid). In this study, the fluoroquinolone finafloxacin was compared to co-trimoxazole as a post-exposure prophylactic in a murine model of inhalational glanders. BALB/c mice were exposed to an aerosol of *B. mallei* followed by treatment with co-trimoxazole or finafloxacin initiated at 24 h post-challenge and continued for 14 days. Survival at the end of the study was 55% or 70% for mice treated with finafloxacin or co-trimoxazole, respectively, however, this difference was not significant. However, finafloxacin was more effective than co-trimoxazole in controlling bacterial load within tissues and demonstrating clearance in the liver, lung and spleen following 14 days of therapy. In summary, finafloxacin should be considered as a promising alternative treatment following exposure to *B. mallei.*

## Introduction

*Burkholderia mallei* is a Gram-negative, non-motile, facultative intracellular pathogen, which is the causative agent of the disease glanders. *B. mallei* is a close phylogenetic relative to *Burkholderia pseudomallei*, which causes the disease melioidosis, both highly infectious by the inhalational route (Cheng et al., 2006; Whitlock et al., 2007). Glanders is prevalent in the Middle East, Asia, Africa and Central and Southern America and is primarily an equine disease (Nguyen et al., 2022). Infection in humans is often associated with occupational exposure to infected animals, e.g., veterinarians, butchers and farmers (Van Zandt et al., 2013; Nguyen et al., 2022), however, laboratory acquired infections have also been reported (Dvorak and Spickler, 2008). Clinical presentation in humans can be acute or chronic dependent on the route of infection (Whitlock et al., 2007) and can present as cutaneous (farcy) or nasal-pulmonary (glanders) (Dvorak and Spickler, 2008).

Human cases of glanders are rare, however, *B. mallei* infected horses were utilized as a biological weapon, in both World War I and World War II (Hawley and Eitzen, 2001). It is still considered to be a potential biothreat pathogen, listed by the U.S. Centers for Disease Control and Prevention as a category B biological agent (Centers for Disease Control and Prevention, 2000). Like *B. pseudomallei, B. mallei* has a high mortality rate if left untreated, with death occurring within 7–10 days of onset of disease. Mortality is up to 50% even with appropriate antibiotic treatment (Nguyen et al., 2022).

Despite continued research, there are currently no licensed vaccines available for melioidosis or glanders (Titball et al., 2017) and due to glanders cases being relatively rare in humans, there is limited information available on antibiotic therapy. *B. pseudomallei* is inherently resistant to many antibiotics (Dance, 2014), whereas fluoroquinolones, macrolides, tetracyclines and aminoglycosides have been shown to have good *in vitro* activity against *B. mallei* (Kenny et al., 1999; Heine et al., 2001). However, due to the lack of glanders cases reported these antibiotics have not been assessed for efficacy in humans. Sulfadiazine was used to successfully treat six laboratory-acquired infections that occurred at Fort Detrick, in addition to a further two cases that were not acquired within a laboratory (Van Zandt et al., 2013). In the most recent case of laboratory-acquired glanders, the patient was successfully treated with imipenem and doxycycline intravenously for 1 month followed by oral azithromycin and doxycycline for 6 months (Srinivasan et al., 2001). The current recommendation for the treatment of human glanders is to use the same regimens that are used for the treatment of melioidosis (Dance, 2014) (parenteral ceftazidime or meropenem for 10–14 days for the acute phase of disease followed by co-trimoxazole delivered orally for 12–20 weeks for the oral eradication phase) (Chetchotisakd et al., 2014). It is suggested that due to the similarity to melioidosis, post-exposure prophylaxis (PEP) in the event of a known exposure to *B. mallei*, should be the same regimens used for *B. pseudomallei.* In the UK the current recommended PEP following exposure to *B. pseudomallei* is co-trimoxazole (960 mg delivered orally twice daily) for 7 days, which is informed from animal studies (Health Protection Agency, 2008). Other international recommendations suggest that co-trimoxazole should be administered for 21 days (960 mg tablets, 2 every 12 h) (Dance, 2014). Due to the limited data available on treating glanders in humans, the evaluation of new treatments for efficient PEP and treatment of *B. mallei* infections is required.

Finafloxacin is a novel C-8-cyano-fluoroquinolone containing a unique chiral C7 substituent, with enhanced activity under acidic conditions which are often found at the site of infection and where other fluoroquinolones, including ciprofloxacin, are less active (Emrich et al., 2010; Higgins et al., 2010). Therefore, finafloxacin may exhibit advantages over other fluoroquinolones in the treatment of bacteria that inhabit acidic cellular organelles, for example the phagosomes or phagolysosomes (Lemaire et al., 2011). Finafloxacin is being developed by MerLion Pharmaceuticals Gmbh for the treatment of serious infections with acidic foci and has obtained approval by the US FDA and Health Canada as a topical medication for the treatment of acute otitis (Emrich et al., 2010; Bartoletti et al., 2015; McKeage, 2015). Finafloxacin has been evaluated in preclinical and clinical studies that have shown that finafloxacin has increased bactericidal activity at infection-relevant acidic pH against intracellular *Legionella pneumophila*, ciprofloxacin resistant strains of *Escherichia coli* and *Acinetobacter baumannii*, and other Gram-negative and Gram-positive bacterial species (Emrich et al., 2010; Higgins et al., 2010; Dalhoff et al., 2011; Lemaire et al., 2011; Stubbings et al., 2011; Vente, 2015). Furthermore, we have previously demonstrated that finafloxacin has *in vitro* activity and *in vivo* efficacy against a range of biothreat pathogens including *Yersinia pestis, B. pseudomallei, Francisella tularensis, Bacillus anthracis*, and *Coxiella burnetii* (Barnes et al., 2017, 2019, 2021; Hartley et al., 2021).

The aim was to determine the *in vitro* activity of finafloxacin against *B. mallei*, in both neutral and acidic conditions. Furthermore, to determine if PEP with finafloxacin can confer protection and also prevent a persistent infection from being established, which is often seen with *Burkholderia* infections (Currie et al., 2000). The *in vivo* efficacy of orally administered finafloxacin (as PEP) against an infection with *B. mallei* was investigated in a murine model of inhalational glanders and compared to co-trimoxazole, a component of the treatment regimen of human melioidosis and glanders.

## Materials and methods

### Bacteria

All bacteriological procedures were carried out in a Class III microbiological safety cabinet or a Class III half suit rigid isolator within an Advisory Committee on Dangerous Pathogens (ACDP) Containment Level 3 laboratory.

### *In vitro* assays

A loop of a frozen glycerol stock of *B. mallei* strain 23344 was added into 10 mL of Cation Adjusted Mueller Hinton broth (CAMHB) and incubated at 37^°^C, shaking at 180 rpm for 18 h.

### *In vivo* study

*B. mallei* was cultured onto L agar + 5% glycerol plates (LAG) and incubated at 37^°^C for 48 h. A loopful of bacteria was then inoculated into 10 mL of Luria Bertani + 5% glycerol (LBG) broth and the OD_590_ nm adjusted to 0.35 ± 0.01. 1 mL of the adjusted culture was inoculated into LBG broth and incubated at 37^°^C, shaking at 180 rpm for 50 h.

### Animals

Female BALB/c mice aged 8–10 weeks were purchased from Charles River Laboratories (UK) and randomized into cages of 5 within a Class III half suit rigid isolator in an ACDP level 3 laboratory. Mice had a 7 day acclimatization period before any procedures were performed and they had free access to water and rodent diet (Harlan Teklad, UK) throughout the study.

### Antibiotics

Finafloxacin was supplied by MerLion Pharmaceuticals GmbH. Sulfamethoxazole and trimethoprim were purchased from Sigma Aldrich (UK) for the *in vitro* assays and an oral suspension of co-trimoxazole (Septrin) was purchased from GlaxoSmithKline (UK) for use in the *in vivo* studies.

For the *in vitro* assays, working concentrations of finafloxacin at 10 mg/mL were prepared by adding 100 mg of antibiotic to 9 mL of sterile water and 1 mL of 1 M sodium hydroxide (NaOH). Co-trimoxazole (10 mg/mL) was prepared by adding 16.5 mg of trimethoprim to 4.985 mL distilled water and 15 μL acetic acid, and 83.5 mg of sulfamethoxazole was added to 4.5 mL of distilled water and 0.5 ml of 1M NaOH, and the 2 components mixed. Bacteria grown in the equivalent concentration of sodium hydroxide used to prepare the antibiotics was included as a control.

For the *in vivo* studies a 15 mg/mL solution of finafloxacin was prepared by adding 2.1 mL of 0.01 M Tris buffer to 44 mg of finafloxacin powder (containing 37.5 mg of active ingredient). 200 μL of 1 M NaOH was added to dissolve the antibiotic followed by 200 μL of 0.01 M hydrochloric acid. The pH of the resulting solution was pH 8; a new solution was prepared for each time point. Co-trimoxazole was diluted in PBS to obtain a concentration of 1.56 mg per 50μL dose.

### Minimum inhibitory concentration

Minimum inhibitory concentration (MICs) for finafloxacin and co-trimoxazole were determined for *B. mallei* using the broth micro-dilution method in accordance with the Clinical Laboratory Standards Institute (CLSI) guidelines (Clinical and Laboratory Standards Institute, 2006). Assays were performed in 96 well micro-titer plates in CAMHB adjusted to pH 5 or pH 7, with antibiotic concentrations in the range of 64–0.03 μg/mL, and bacteria at a final concentration of approximately 5 × 10^5^ CFU/mL. *Escherichia coli* ATCC25922 was included as a quality control strain. All assays were performed in triplicate.

### Minimum bactericidal concentration

Minimum bactericidal concentration (MBCs) for finafloxacin and co-trimoxazole were determined by plating 100 μL aliquots of the MIC dilutions showing no visible growth onto LAG in triplicate and incubating at 37^°^C for 48 h. The MBC was recorded as the lowest concentration of antibiotic that killed 99.9% of the bacteria in the original inoculum (Clinical and Laboratory Standards Institute, 2006).

### Generation of aerosolized bacterial challenge

*B. mallei* was aerosolized using a 3-jet Collison nebuliser, containing a volume of 15 mL of bacteria, controlled and conditioned to an average of 71.3% (range 69.25–73.4%) relative humidity, by an AeroMP platform system (Biaera Technologies, Hagerstown, MD, USA) (Hartings and Roy, 2004). Animals were exposed (nose only) to the aerosol for a total of 10 min, with sampling achieved for 1 min at the mid-point of the challenge (4.5–5.5 min) using an all-glass impinger (AGI-30; Ace Glass, Vineland, NJ, USA) containing 10 mL PBS.

The calculated, presented challenge dose was determined using the bacterial enumerations from the aerosol samples and Guyton’s formula for the respiratory volumes of laboratory animals (20 mL min^–1^) (Guyton, 1947). A retained dose of 40% of the presented dose was also calculated (Harper and Morton, 1953).

### Antibiotic regimens

Antibiotic regimens were determined previously by matching to the pharmacokinetic (PK) parameter that correlated with efficacy *in vivo* and a human equivalent dose. For finafloxacin, the AUC/MIC over 24 h period in a human dose was determined and resulted in a dose in BALB/C mice of 37.5 mg/kg every 8 h (Barnes et al., 2021). For co-trimoxazole, the time above MIC achieved in humans was matched giving a dose of 78 mg/kg every 12 h (unpublished data).

### *In vivo* efficacy study

Therapy was initiated at 24 h post-challenge. Groups of 20 mice were administered finafloxacin (37.5 mg/kg) in a 50 μL oral dose *via* pipette every 8 h or co-trimoxazole (78 mg/kg) in a 50 μL oral dose *via* pipette every 12 h. Control groups of infected mice were administered 50 μL diluent (consisting of Tris, sodium hydroxide and hydrochloric acid, adjusted to pH 8) orally *via* pipette every 8 h. All treatment regimens were continued for 14 days. Co-trimoxazole was diluted in PBS to obtain a concentration of 1.56 mg per 50μL dose.

Mice were weighed daily and clinical signs of disease were recorded twice daily up to the point that the experiment was terminated at day 65 post-challenge. These clinical signs were recorded based on observed changes to the animal’s behavior and condition (including piloerection, hunching and changes to mobility and respiration). Mice were euthanized *via* a Schedule 1 procedure once they had reached their humane endpoint.

Additionally, following 1 day of antibiotic treatment and at the end of the treatment regimen 5 mice from each group were culled. Post mortems were performed and the spleen, liver and lungs harvested, weighed, and homogenized in 1 mL of sterile PBS. A 10-fold serial dilution was performed and 100 μL aliquots were plated onto LAG in duplicate. The agar plates were incubated for 48 h at 37^°^C and bacteria enumerated to determine the level of colonization in the organs. At the end of the study all surviving animals were euthanized, post-mortems performed, and organs harvested as described above. In addition, brain samples were also collected at this time point.

### Statistical analysis

All graphs were produced using the program GraphPad PRISM V8.0.1. Statistical analysis was performed using IBM, SPSS 27.0. Survival data was analyzed using log rank tests. The Bonferroni’s correction was used to correct for family-wise error. The bacterial colonization data consisted of multiple zeros necessitating a count-based statistical model. The estimated bacterial load per gram of tissue was used for analysis. To compare treatment groups, a negative binomial generalized linear model with a log link was constructed. Animal weight data was converted to a percentage and compared to their weight at time of infection. This data was analyzed using a repeated measures linear model. This model was divided into specific comparisons and the Bonferroni’s correction was used to correct for family-wise error. Model suitability against assumptions were considered by using diagnostic plots (residual plots and half QQ-plots). The clinical scores data was analyzed using mixed generalized Poison models. Where pairwise comparisons were performed, the Bonferroni’s method was used to correct for family-wise error. The interaction term for the treatment group and time was considered the meaningful output for comparing the accumulation of clinical signs of disease.

## Results

### Finafloxacin is more active than co-trimoxazole *in vitro*

The activity of finafloxacin against *B. mallei* was compared with co-trimoxazole by MIC and MBC at pH 5 and pH 7. *E. coli* ATCC25922 was included as a quality control strain and was within the expected range. The MIC for finafloxacin was 2 and 0.5 μg/mL at pH 5 and pH 7, respectively (Table 1). In comparison, the MIC for co-trimoxazole was much higher at both pHs (32 and 8 μg/mL at pH 5 and pH 7, respectively). In MBC assays finafloxacin demonstrated a similar level of bactericidal activity at both pHs (2 and 1 μg/mL at pH 5 and pH 7, respectively) compared to co-trimoxazole, which did not demonstrate bactericidal activity up to a concentration of 64 μg/mL at either pH.

**TABLE 1:** The MICs and MBCs for finafloxacin and co-trimoxazole for *B. mallei* strain 23,344.

Antibiotic	MIC (μg/mL)		MBC (μg/mL)	
	pH 5	pH 7	pH 5	pH 7
Finafloxacin	2	0.5	2	1
Co-trimoxazole	32	8	>64	>64

Assays were performed in CAMHB adjusted to pH 5 or pH 7 in triplicate.

### Finafloxacin offers protection against *Burkholderia mallei in vivo*

The efficacy of finafloxacin was compared to co-trimoxazole and control animals treated with the vehicle (Tris buffer) in BALB/c mice. Mice were challenged with a mean retained dose of 1.52 × 10^4^ CFU (range 8.27 × 10^3^ – 2.08 × 10^4^ CFU) equating to approximately 44 median lethal doses (MLDs, 1MLD = approximately 343 CFU) by the inhalational route. One mouse in the finafloxacin treatment group turned in the tube during the aerosol delivery, meaning that the challenge dose could not be assured for this animal. Therefore, it was removed from the study at this point, leaving 9 mice in this group.

**FIGURE 1:**
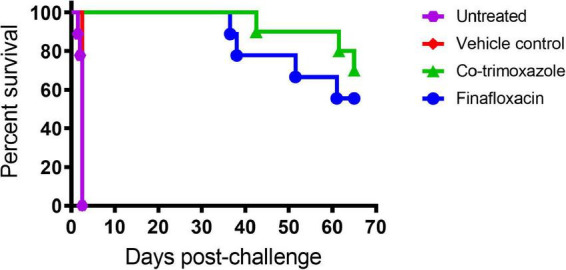
The percentage survival of mice following challenge with aerosolized *B. mallei*. Mice were challenged with a mean retained dose of 1.52 × 10^4^ CFU of *B. mallei* by the inhalational route and treated with finafloxacin (37.5 mg/kg) every 8 h or co-trimoxazole (78 mg/kg) every 12 h by the oral route. Control animals received vehicle by the oral route every 8 h. Regimens were initiated at 24 h post-challenge and continued for 14 days.

At 24 h post-challenge (at treatment initiation), mice were presenting with minor clinical signs of disease (with an average clinical score of 1). All control animals (untreated or treated with the vehicle) succumbed to infection by day 4 post-challenge, demonstrating that the *B. mallei* challenge was lethal (Figure 1).

Both antibiotics offered significant protection in comparison to animals receiving the vehicle control or no treatment (*p* < 0.004). There was no difference between the untreated mice and the mice that received the vehicle (*p* = 0.500). Survival in mice treated with finafloxacin or co-trimoxazole was 55 or 70%, respectively, however, these differences were not significantly different from each other (*p* > 0.999).

### Mice treated with finafloxacin had reduced levels of bacteria in tissues following 1 day of treatment

**FIGURE 2:**
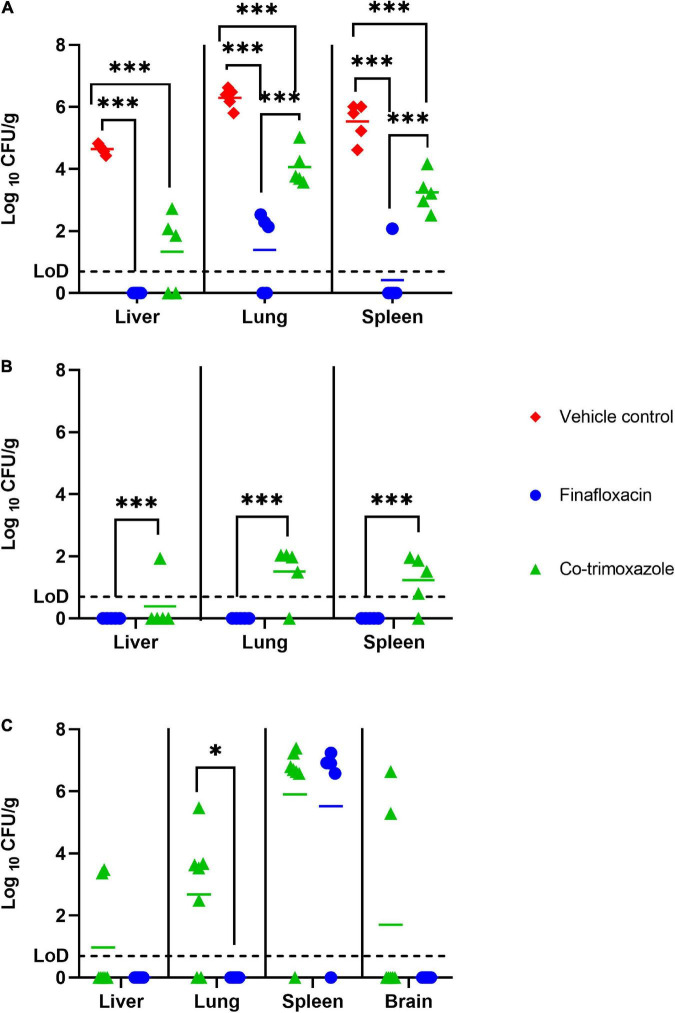
The bacterial load in organs of mice challenged with *B. mallei* and treated with antibiotics. Bacterial counts (CFU/g) in the spleen, livers and lungs of mice following 1 day of antibiotic treatment **(A)**, 14 days of antibiotic treatment **(B)** or at the end of study day 65 (brains also harvested) **(C)**. Therapy was initiated at 24 h post-challenge and continued for 14 days. Treatment was with finafloxacin (37.5 mg/kg) every 8 h or co-trimoxazole (78 mg/kg) every 12 h delivered by the oral route. LoD, limit of detection ***p < 0.001, *p < 0.05.

Five mice per group (treated with finafloxacin, co-trimoxazole or the vehicle control) were euthanized at 48 h post-challenge, when they had received one full day of treatment. Following 1 day of therapy, the mice treated with finafloxacin or co-trimoxazole had lower levels of *B. mallei* in the spleen, liver and lungs compared to the vehicle treated controls (*p* < 0.001) (Figure 2A). Furthermore, the mice treated with finafloxacin had a reduced bacterial load in the spleen, lungs and liver in comparison to the co-trimoxazole treated mice (*p* < 0.001). 100, 40, and 80% of the livers, lungs and spleens harvested from animals treated with finafloxacin were clear of colonizing bacteria. All organs harvested from mice treated with co-trimoxazole were colonized apart from 40% of the liver samples. Following incubation of the remaining homogenate, one mouse treated with finafloxacin was colonized in the lung (other organs clear).

### Mice that received finafloxacin therapy were clear of colonizing bacteria in tissues at the end of the treatment period

Five mice per group (treated with finafloxacin or co-trimoxazole) were euthanized at the end of the 14 days of antibiotic therapy. Mice treated with finafloxacin had lower levels of *B. mallei* in the spleen, liver and lungs when compared to mice treated with co-trimoxazole (*p* < 0.001). Specifically, no bacteria was detected in the organs harvested from mice treated with finafloxacin. Whereas *B. mallei* was detected in 20, 80, and 80% of livers, lungs and spleens harvested from mice treated with co-trimoxazole (Figure 2B).

### Fewer tissues from mice treated with finafloxacin were colonized with *Burkholderia mallei* at the end of the study

The study was terminated at day 65 post-challenge. It was not possible to statistically model the bacterial load data at the end of study, as due to the survivor biased nature of the data set, repeated measure linear models were unable to function with missing data. At the end of the study no *B. mallei* was detected in the liver, lungs or brain of mice treated with finafloxacin (Figure 2C). 29, 71, and 29% of livers, lungs and brains harvested from mice treated with co-trimoxazole were colonized. However, 80 and 86% of spleens harvested from mice treated with either finafloxacin or co-trimoxazole, respectively, were heavily colonized with bacteria. In the spleens where bacteria was detected, splenomegaly with multiple abscesses was also observed.

### Mice treated with finafloxacin lost more weight than those treated with co-trimoxazole

**FIGURE 3:**
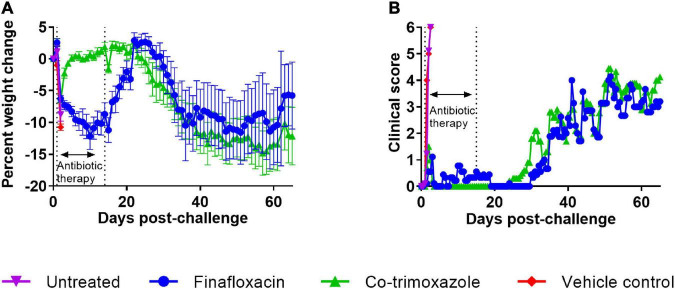
The weight loss and clinical scores of mice challenged with *B. mallei* and treated with antibiotics. Mice were challenged with a mean retained dose of 1.52 × 10^4^ CFU of *B. mallei*. Therapy was initiated at 24 h post-challenge with finafloxacin (37.5 mg/kg) every 8 h or co-trimoxazole (78 mg/kg) every 12 h by the oral route. Control animals received vehicle by the oral route. Treatment was continued for 14 days. The mean percentage weight loss when compared to the original bodyweight is shown for each treatment group **(A)** and the mean clinical score **(B)**.

The bodyweights and clinical scores were recorded and are shown in Figure 3. The control mice rapidly lost weight until they succumbed to infection. The mice treated with co-trimoxazole initially lost weight but regained this following the initiation of treatment and remained at baseline until day 21 post-challenge, when there was a gradual decline in weight, accompanied with an increase in clinical score (Figures 3A,B). The mice treated with finafloxacin showed a decline in weight for the duration of the treatment but then gained weight back to baseline following cessation of treatment with minor clinical signs recorded. A gradual decline in weight was observed from day 30 post-challenge, which was also accompanied with an increase in clinical signs.

Statistical analysis of animal weight was performed using two approaches. One approach investigated differences between the four groups (before the untreated animals succumbed to infection). This analysis used data between 1 and 2 days post-challenge and indicated that different treatment groups exhibited different weight change profiles (*p* < 0.001). Post-tests were used to determine any difference between treatment groups. Both antibiotic treatments resulted in mice losing less weight when compared to the vehicle groups (*p* < 0.001), however, (using this limited subset of the data) there was only evidence for finafloxacin providing a benefit compared to the naïve mice (*p* = 0.028). There was no evidence for a difference between the control groups (*p* = 0.125). In the second approach, only antibiotic treated animals that were not culled for experimental purposes were included. This analysis ran from 1 to day 36 post-challenge (when antibiotic treated animals started succumbing to disease). This analysis confirmed that co-trimoxazole was better at preventing weight loss (*p* < 0.004).

Progression of disease was recorded using a score-based method (Figure 3B). Differences between treatment groups were not obvious so statistical modeling methods were used to interrogate the data. First, the clinical scores data was analyzed between days 0 and 2.5 post-challenge to compare the antibiotic treated groups to the “untreated” groups. Both antibiotics protected against the development of clinical signs of disease when compared to either control group (*p* < 0.004) in all cases. A further analysis was performed between days 0 and 36 post-challenge in the antibiotic treated groups. Here we observed a very small reduction, in the clinical signs recorded in the animals treated with finafloxacin (*p* < 0.004). While these reductions are unlikely to have any meaningful effect on disease, they are counter to the observations regarding the loss of weight data.

## Discussion

Although *B. mallei* primarily infects solipeds, human infections can occur as a consequence of occupational exposure and are almost always fatal without antibiotic therapy. Current information on antibiotic resistance in *B. mallei* is limited, but it is believed to be high, however, MICs have been shown to be lower than those for *B. pseudomallei* (Thibault et al., 2004). Even when treatment is administered, glanders can be lethal, therefore it is essential to assess new therapeutics for use as both PEP and treatment.

Finafloxacin has proven potent bactericidal activity for a spectrum of Gram-positive and Gram-negative organisms, this importantly includes panels of both *B. pseudomallei* and *B. mallei* strains (Barnes et al., 2019). In contrast to data generated with other bacterial species, the MIC of finafloxacin is slightly higher at pH 5 at 2 μg/mL, compared to 0.5 μg/mL at pH 7 for *B. mallei* strain 23344. It may be that this is a strain specific issue as in previous studies have shown that the MIC_90_ for a panel of *B. mallei* strains was equivalent at both pHs and the MIC_50_ was lower at pH 5 compared to pH 7 for finafloxacin (Barnes et al., 2019). Even though the MIC was higher at pH 5 the MBC was also shown to be 2 μg/mL. Therefore although activity appears to be slightly reduced at pH 5, it still remains bactericidal at relatively low concentrations. Further evidence for this was seen in time kill assays, where at 4 times the MIC, finafloxacin was bactericidal at pH5 but not at pH7 (data not shown). MICs determine the inhibitory activity at a specific time point whereas time kill assays provide information on killing activity over a time course, which may be a more relevant for difficult to treat infections such as those caused by *B. pseudomallei* and *B. mallei.* This highlights the importance of utilizing different *in vitro* assays to determine the efficacy of an antibiotic and understanding the limitations of each use.

By 48 h following an inhalational challenge in BALB/c mice, *B. mallei* had disseminated from the lungs to the liver and spleen, which were both heavily colonized (10^4^–10^6^ CFU/g). This resembles acute disease that is seen in humans, which is dependent on the route of infection but even inoculation of bacteria into the skin generally results in pneumonia and septicemia (Van Zandt et al., 2013). A similar disease progression is also observed in the marmoset model of sub-cutaneous glanders, where all animals developed multifocal necrotizing pneumonia (Nelson et al., 2014).

Although no difference in protection was observed between finafloxacin and co-trimoxazole, finafloxacin treatment appeared to be better at controlling the level of bacteria in organs and the development of clinical signs of disease. Multiple studies have demonstrated the *in vitro* activity and *in vivo* effectiveness of finafloxacin, suggested to be due to finafloxacin entering the cell very rapidly, accumulating to high levels and remaining within the cell, effluxing out very slowly (Chalhoub et al., 2020). One day of treatment with finafloxacin resulted in bacterial clearance in the liver and reduced levels of colonization in the spleen and lung when compared to mice that received co-trimoxazole, that were all colonized in the spleen and lungs. Furthermore, at the end of the treatment period, *B. mallei* was not detected in the liver, spleen or lungs of the mice treated with finafloxacin. Whereas all of the co-trimoxazole treated mice were colonized in at least one organ. The absence of bacteria in the organs suggests that treatment with finafloxacin provides clearance of the bacterial infection. However, the bacterial load data from the surviving mice at day 65 revealed that all of the mice, except one, were colonized with bacteria in the spleen. Finafloxacin treatment did, however, prevent the recrudesce of disease in the lungs and liver. Whereas in mice that received treatment with co-trimoxazole 6 out of the 7 surviving mice had bacteria in the spleen and were colonized with a high concentration of bacteria in at least one other organ.

Co-trimoxazole was able to provide protection against bodyweight loss. Previous work has shown that animals treated with the same regimen of finafloxacin in the absence of infection do not show weight loss during the treatment period (unpublished data). Whereas the data in this study suggests that it is the combination of the treatment and the infection causing this weight loss. Once treatment with finafloxacin ceases, the mice quickly regain weight back to their baseline weight providing further evidence that the weight loss observed is due to the combination of the infection and the antibiotic treatment. Other possibilities could include the release of Pathogen Associated Molecular Patterns (PAMPs), by the bacteria following finafloxacin treatment. However, if this were the case, we would have expected to see an increase in the clinical signs of infection recorded in this group. It is also possible that this drug, in combination with the subclinical disease, differentially affected the gut microbiome of the animals, which is known to take place in humans with other fluoroquinolones (Carbon, 2001). Oral antibiotic treatment in mice has been shown to modulate the microbiome in the gut resulting in weight loss, but this is dependent on the regimen and antibiotic used and not all antibiotics have this effect (Miao et al., 2020; Bongers et al., 2022). This may explain why the mice treated with co-trimoxazole did not lose weight during treatment whereas the mice treated with finafloxacin did. This requires further investigation.

Although limited, other studies have investigated alternative antibiotics for the treatment of glanders. One study determined the efficacy of moxifloxacin, azithromycin or co-trimoxazole against an inhalational infection with 1,000–3,000 CFU of *B. mallei* (Waag, 2015). Of most relevance to this manuscript is the efficacy offered by co-trimoxazole (sulfamethoxazole 200-mg/kg)-trimethoprim (40-mg/kg; Q12 h; i.p.) initiated on day 5 post-challenge for 5 or 10 days. Low levels of protection (20 and 30% for 5 or 10 days of treatment, respectively) were offered by co-trimoxazole in this study and spleens were colonized, however, the treatment was only initiated 5 days into the infection.

Another study investigated ceftazidime (100 mg/kg/day) and levofloxacin (20 mg/kg/day) (Judy et al., 2009) delivered by the intraperitoneal route as post-exposure treatment, 24 h following an intranasal challenge with *B. mallei.* All mice survived until the study terminated at day 34 post-challenge, however, the lungs and spleens were colonized with bacteria (Estes et al., 2010).

In the study detailed in this manuscript, both finafloxacin and co-trimoxazole offered complete protection until day 36 and 42 post-challenge for mice treated with finafloxacin and co-trimoxazole, respectively. Clearly, both antibiotics were able to treat the acute form of glanders, with some mice relapsing weeks after the cessation of treatment. It is possible that the organs appeared clear from colonizing bacteria, however, there are bacterial cells present in a “persister” or “differentially culturable” state that cannot be cultured on solid agar, known to be an issue in infections with *Mycobacterium tuberculosis* (Gordhan et al., 2021) and *B. pseudomallei* (Auty et al., 2022). Further investigation to prove or disprove this hypothesis would be advantageous in designing new treatment regimens for glanders.

This study has demonstrated that treatment of an infection with *B. mallei* with finafloxacin is comparable to co-trimoxazole with regards to survival; however, finafloxacin demonstrates a benefit in the control of bacterial load in tissues. This could be an important factor with clinical relevance and suggests that further studies are needed. These could include the investigation of the effect of both of these parameters when the window of opportunity is extended for finafloxacin used as a monotherapy and also as a combination therapy, demonstrated to be a successful approach with *B. pseudomallei* (Barnes et al., 2022).

## Data availability statement

The original contributions presented in this study are included in the article/Supplementary material, further inquiries can be directed to the corresponding author.

## Ethics statement

This animal study was reviewed and approved by the DSTL AWERB.

## Author contributions

KB, MB, AV, and SH conceived the concept and designed the experiments detailed in this manuscript. KB, MB, CD, and MR conducted the experiments. KB, CD, MB, and TL performed the data analysis. KB, TL, and SH wrote the manuscript. All authors reviewed the draft and approved the manuscript for publication.
